# Supplementary material for: In Vitro Investigation of the Effects of Various Reducing Agents on Dentin Treated with Hydrogen Peroxide
Source: Polymers (Basel). 2024 May 23;16(11):1473. doi: 10.3390/polym16111473 (PMC11174467; doi:10.3390/polym16111473)
Supplement: Supplementary file 1 [file polymers-16-01473-s001.zip › polymers-2998229-supplementary.pdf]

Supplementary Figures for the study titled "In vitro investigation of the effects of various reducing agents on dentin treated with hydrogen peroxide" in Polymers.

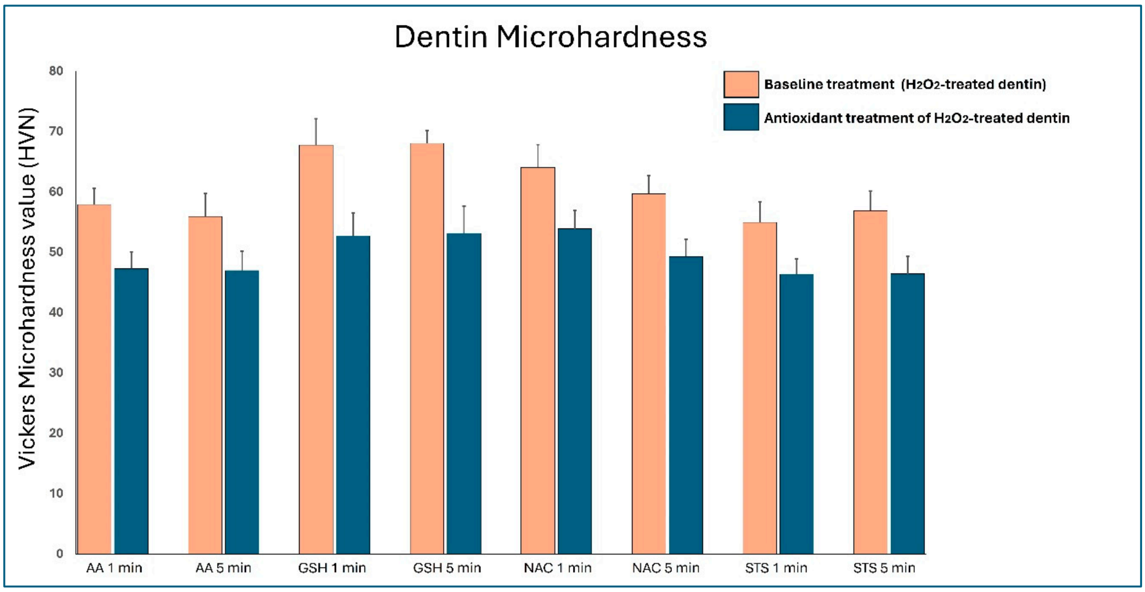

Figure S1. The average microhardness values for the baseline treatment (dentin treated with H_2O_2 for 15 min) in each group, along with the microhardness values after treatment of this substrate with the antioxidants for 1 or 5 min. H_2O_2: hydrogen peroxide, AA: ascorbic acid, GSH: reduced glutathione, NAC: *n*-acetylcysteine, STS: sodium thiosulfate.

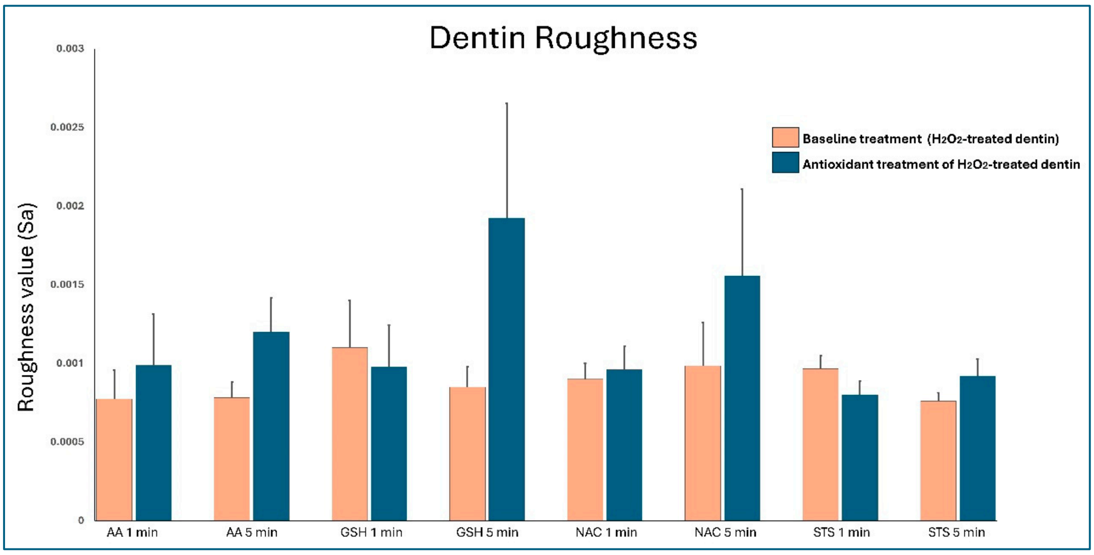

Figure S2. The average roughness values for the baseline treatment (dentin treated with H_2O_2 for 15 minutes) in each group, along with the roughness values after treatment of this substrate with the antioxidants for 1 or 5 min. H_2O_2: hydrogen peroxide, AA: ascorbic acid, GSH: reduced glutathione, NAC: *n*-acetylcysteine, STS: sodium thiosulfate.
